# Influence of various temperatures, seed priming treatments and durations on germination and growth of the medicinal plant *Aspilia africana*

**DOI:** 10.1038/s41598-022-18236-2

**Published:** 2022-08-19

**Authors:** Denis Okello, Richard Komakech, Roggers Gang, Endang Rahmat, Yuseong Chung, Francis Omujal, Youngmin Kang

**Affiliations:** 1 Herbal Medicine Resources Research Center, Korea Institute of Oriental Medicine (KIOM), 111 Geonjae-ro, Naju-si, Jeollanam-do 58245 Republic of Korea; 2 Korean Convergence Medicine Major, University of Science and Technology (UST), Daejeon, Republic of Korea; 3 Natural Chemotherapeutics Research Institute (NCRI), Ministry of Health, P.O. Box 4864, Kampala, Uganda; 4 National Semi-Arid Resources Research Institute (NaSARRI), P.O. Box 56, Soroti, Uganda

**Keywords:** Plant sciences, Plant physiology

## Abstract

For millennia, *Aspilia africana* has been used across Africa to treat various diseases including malaria, wounds, and diabetes. In this study, temperature influenced the in vitro germination of *A. africana* with highest final germination percentage (FGP) and germination index (GI) of 65.0 ± 7.64% and 2.26 ± 0.223, respectively, at 19.8 °C. Priming seeds with H_2_O, KNO_3,_ and GA_3_ (gibberellic acid 3) improved both in vitro germination and ex vitro emergence of *A. africana* seeds. Seed priming with $1.44 \times 10^{ - 3 }$ M GA_3_ produced overall highest in vitro FGP (from 90.0 ± 4.08% to 100 ± 0.00%) and GI (from 2.97 ± 0.385 to 3.80 ± 0.239) across all priming durations. Seeds primed with KNO_3_ had better germination parameters for 6 and 12 h compared to 18 and 24 h. Furthermore, the highest in vitro FGP (100 ± 0.00%) was observed in seeds primed for 12 h with $1.44 \times 10^{ - 3 }$ M GA_3_. Ex vitro* A. africana* seed emergence was significantly enhanced by GA_3_ priming. Priming *A. africana* seeds with H_2_O, KNO_3_, and GA_3_ improved their growth after 3 months, with the overall best growth for seeds primed with $2.89 \times 10^{ - 4 }$ M GA_3_. Seed priming of *A. africana* is a feasible approach for improving germination and seed emergence, and enhancing plant growth.

## Introduction

*Aspilia africana* (Pers.) C. D. Adams, also known as wild sunflower, hemorrhage plant, or African iodine plant, has been used for millennia to treat several diseases across many countries in Africa^1,2^. Diseases and health conditions treated using *A. africana* include malaria, osteoporosis, tuberculosis, febrile headaches, diabetes, stomach ache, cough, rheumatic pains, measles, diarrhea, ear infections, wounds, sores, gastric ulcers, gonorrhea, and stings from bees, wasps and scorpions^3–5^. In a recent study, Niyonizigiye, et al.^6^ demonstrated the plant’s anti-cancer activity. The biological activity of the plant is attributed to its richness in secondary metabolites such as phenolic compounds (including chlorogenic acid and gallic acid), flavonoids (e.g., quercetin), tannins, saponins, and terpenes (such as caryophyllene, phytol, and pinene)^1,5^. *A. africana,* although indigenous to Eastern African counties, inhabits forest zones of tropical Africa and the savanna^5,7^.

Apart from soil moisture, the most vital abiotic factor that greatly influences seed germination is temperature^8^. The effects of temperature on germination vary across species or even among the seeds of a species from different provenances^8,9^. Temperature not only influences germination but also greatly regulates growth and development in plants^10,11^. The temperature at which germination percentage is highest is termed as the optimum temperature and this varies from one species to another^8,10^. Understanding the emergence and germination responses of plants to temperature is critical as it not only provides a basis for temperature tolerance identification but also provides an understanding of optimal climatic conditions for germination and successful establishment of plants^10^ in addition to assisting in model construction to predict developmental processes^12^.

Seed priming is a widely used low-cost pre-sowing strategy for improving imbibition and inducing DNA repair processes and antioxidant responses linked to pre-germinative metabolism without radicle protrusion^13–15^. After the seeds are primed, they are dehydrated, stored, or commercialized^13^. Different priming techniques, such as osmopriming, hydropriming, chemical priming, hormonal priming, and nutrient priming have been employed to improve seed germination and crop yield^16^. Seed priming enhances germination and results in fast and uniform emergence of plants^13^. Furthermore, priming increases tolerance of plants to abiotic and biotic stresses, greatly improving plant population density and performance^13^.

*A. africana* is not only a plant of great cosmetic and pharmaceutical interests^5,7^ but is also highly browsed by domestic animals such as cattle, goats, rabbits, and sheep^17^. A recent study by Okello, et al.^18^ on the effects of different commercial soils on the germination of *A. africana* indicated a very low plant germination rate. In this study, we investigated the effects of temperature and seed priming conditions on the germination of *A. africana.* This contributes to improving seed germination of this important medicinal plant and its domestication. To the best of our knowledge, this is the first study on the effects of temperature and seed priming on the germination, emergence, and growth of *A. africana*.

## Materials and methods

### Seed material and sterilization

**Figure 1:**
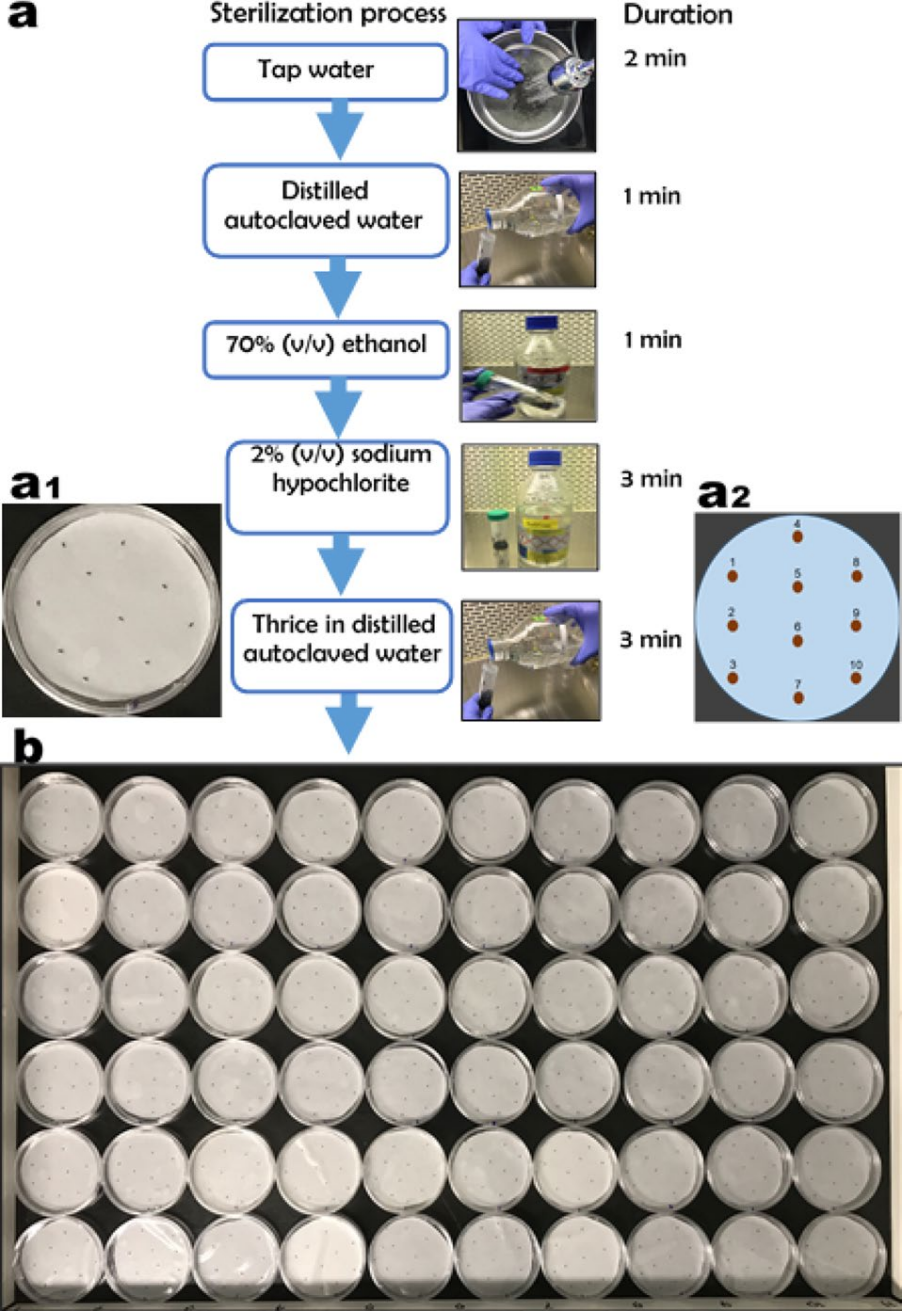
(**a**) Summary of sterilization process of *A. africana* seeds. (**a**1) Arrangement of *A. africana* seeds in a Petri dish (**a**2) Illustration of arrangement of *A. africana* seeds in a Petri dish (**b**) Seeds of *A. africana* in Petri dishes incubated in a thermogradient germinator at different temperatures (1–17.6 °C, 2–18.7 °C, 3–19.8 °C, 4–20.9 °C, 5–22.0 °C, 6–23.1 °C, 7–24.2 °C, 8–25.3 °C, 9–26.4 and 10–27.5 °C.

Mature, dry, and ripe *A. africana* seeds randomly collected with permission from the local authority from at least 50 healthy plants in the wild from Pece, located in Gulu, Uganda, East Africa, and were provided by the Natural Chemotherapeutics Research Institute (NCRI). The seeds were transported to the Korea Institute of Oriental Medicine, Republic of Korea, and stored in a dry room maintained at 25 ± 1 °C until the start of the experiment. A voucher specimen (number KYM-KIOM-2021-1) was deposited at the Korean Herbarium of Standard Herbal Resources (Index Herbarium code: KIOM) at the Korea Institute of Oriental Medicine (KIOM), Herbal Medicine Resources Research Center, Republic of South Korea by Dr. Sungyu Yang. The seeds used in this experimental work were obtained from *A. africana* var*. africana* plants. To limit the tendencies of contamination, the seeds were sterilized as follows: the seeds were first washed under running tap water for 2 min, quickly transferred to a laminar flow cabinet, re-washed with distilled autoclaved water, surface sterilized in 70% (v/v) ethanol for 1 min, followed by 2% (v/v) sodium hypochlorite for 3 min, and then rinsed three times with distilled autoclaved water. Sterilized *A. africana* seeds were used in all in vitro experimental setups. A summary of the sterilization process, in vitro seed arrangement and illustration of the in vitro seed arrangement are shown in Fig. 1a, a1 and a2, respectively. All methods were carried out in accordance with relevant guidelines and regulations.

### Experiment 1: effect of temperature on in vitro germination of *A. africana* seeds

*A. africana* seeds were placed using sterile forceps in crystal-grade polystyrene Petri dishes (100 × 20 mm) containing filter papers moistened with 5 ml distilled water. Each Petri dish contained 10 seeds with six replicates for each temperature, ranging from 17.6 to 27.5 °C with an increment of 1.1 °C, and dishes were placed on a thermogradient germinator chamber under dark conditions (Fig. 1b).

The seeds were monitored for germination every 24 h for 15 d. The Petri dishes with seeds were only exposed to light for a short time during the counting of the germinated seeds. Seeds with a minimum of 2 mm radicle length were counted as germinated. The germination parameters considered for determining the effects of temperature on seed germination of *A. africana* were final germination percentage (FGP), germination index (GI), mean germination rate (MGR), and time required for 50% germination (T_50_). The same parameters were used to investigate the effects of priming treatments and durations on the in vitro and ex vitro germination of *A. africana* seeds. The following formulae were used to calculate the germination parameters: FGP = $\frac{N}{Nt}$ × 100 (*N* is the number of germinated seeds at the final count; *Nt* is the total number of seeds in the Petri dish); GI = ∑ (Gt/Dt) (Gt is the number of germinated seeds on day t, and Dt is time corresponding to Gt in days); and MGR = $\frac{(\sum n)}{{\sum (nt)}}$ (n is the number of newly germinated seeds at time t, and t is the number of days from planting). T_50_ was calculated according to the formula modified from Farooq, et al.^19^, T_50_ = t_i_ + $\frac{{\left[ {\left( {\frac{N}{2} - n_{i} } \right)\left( {t_{j} - t_{i} } \right)} \right]}}{{n_{j} - n_{i} }}$ (*N i*s the final number of germinated seeds, *n*_*i*_ and *n*_j_ are the cumulative number of germinated seeds counted at time *t*_i_ and *t*_j_, respectively, when *n*_i_ < $\frac{N}{2}$ < *n*_j_).

### Experiment 2: effect of priming duration and priming treatments on in vitro germination of *A. africana* seeds

Sterilized *A. africana* seeds were primed at varying times of 6 h, 12 h, 18 h, and 24 h in different priming solutions, such as distilled water (hydro-priming) and potassium nitrate (halo-priming) at different concentrations (0.1, 0.5, and 1 M) and gibberellic acid-3 (GA_3_; hormo-priming) at concentrations of $2.89 \times 10^{ - 5 }$, $2.89 \times 10^{ - 4 }$, and $1.44 \times 10^{ - 3 }$ M. For priming treatments, 60 seeds were placed in 5 ml of each priming solution in crystal-grade polystyrene Petri dishes (100 × 40 mm). Upon completion of the treatment, the primed seeds were rinsed three times with sterile water, blotted, and dried back to their near initial weight at ambient temperature. The seeds were then placed in crystal-grade polystyrene Petri dishes (100 × 20 mm) containing filter paper saturated with autoclaved distilled water. Each Petri dish (100 × 20 mm) contained 10 seeds with five replicates for each treatment. The Petri dishes containing the seeds were kept in a thermogradient germinator chamber maintained at 19.8 °C (determined to be ideal from experiment 1) in darkness. Non-primed *A. africana* seeds were used as a control. Seeds showing signs of fungal contamination were removed from the Petri dishes. The seeds were monitored for germination every 24 h for 15 d and were only exposed to light for a short time during the counting of germinated seeds. Seeds with a minimum of 2 mm radicle length were counted as germinated. The same parameters and formulae used to assess germination in Experiment 1 were used in Experiments 2 and 3.

### Experiment 3: effect of different priming treatments on ex vitro emergence and early growth of *A. africana* plants

To examine the effect of various priming treatments on the ex vitro emergence of *A. africana* seeds, the priming procedure used in Experiment 2 was repeated, except that seeds were treated with the priming solutions for a uniform priming duration of 12 h. The primed seeds were then planted in an autoclaved mixture of horticulture soil (consisting of about 40% mineral soil, 10% organic matter and 50% pore space filled by water and air) containing perlite (Kyungdong ONE Co. Ltd, Republic of Korea), one of the natural volcanic aluminosilicate glasses^20^ and peat pellet soil (Jiffy-7, 33 mm from Jiffy Products International AS, Norway) in a 1:1 ratio (determined as an ideal composition for the growth of *A. africana*^18^) in a plastic planting tray (30 × 25 × 10 cm). Twenty seeds of *A. africana* were planted in each tray at a depth of 1 cm and a distance of 5 cm from each other. Each treatment was replicated three times. The seeds in the soil were watered and the trays were kept in growth chambers maintained at 19.8 ± 1 °C for a 16 h photoperiod. Light intensity was maintained at 33.73 µmol/m2/s using cool white fluorescent tubes. The relative humidity in the growth chamber was maintained at 70%. The seeds in the tray were watered every two days until the end of the experiment. The number of seeds germinated every 24 h was counted and recorded for each treatment until no further emergence occurred. Seeds were counted as emerged when the hypocotyl length was at least 3 mm. The FGP, MGR, T_50_, and GI were calculated. To determine the effects of different priming treatments on the early growth of *A. africana*, seedlings from the differently primed seeds were uniformly re-spaced and allowed to continue growing in the growth chambers, and the growth rates were determined after three months. Each planting tray was carefully immersed in water to soak the soil, enabling easy uprooting of the plants. The roots of the uprooted plants were carefully and thoroughly washed to remove soil particles and debris, and then blotted dry with paper towels. The lengths of roots and shoots of each *A. africana* plant from the different treatments were measured using a meter ruler. The number of leaves and roots of each plant were counted. Fresh weights of the *A. africana* plants from the different treatments were obtained. Thereafter, the plants were oven dried at 60 °C for 48 h and their dry weights were recorded.

## Results

### Effect of temperature on in vitro germination of *A. africana* seeds

**Figure 2:**
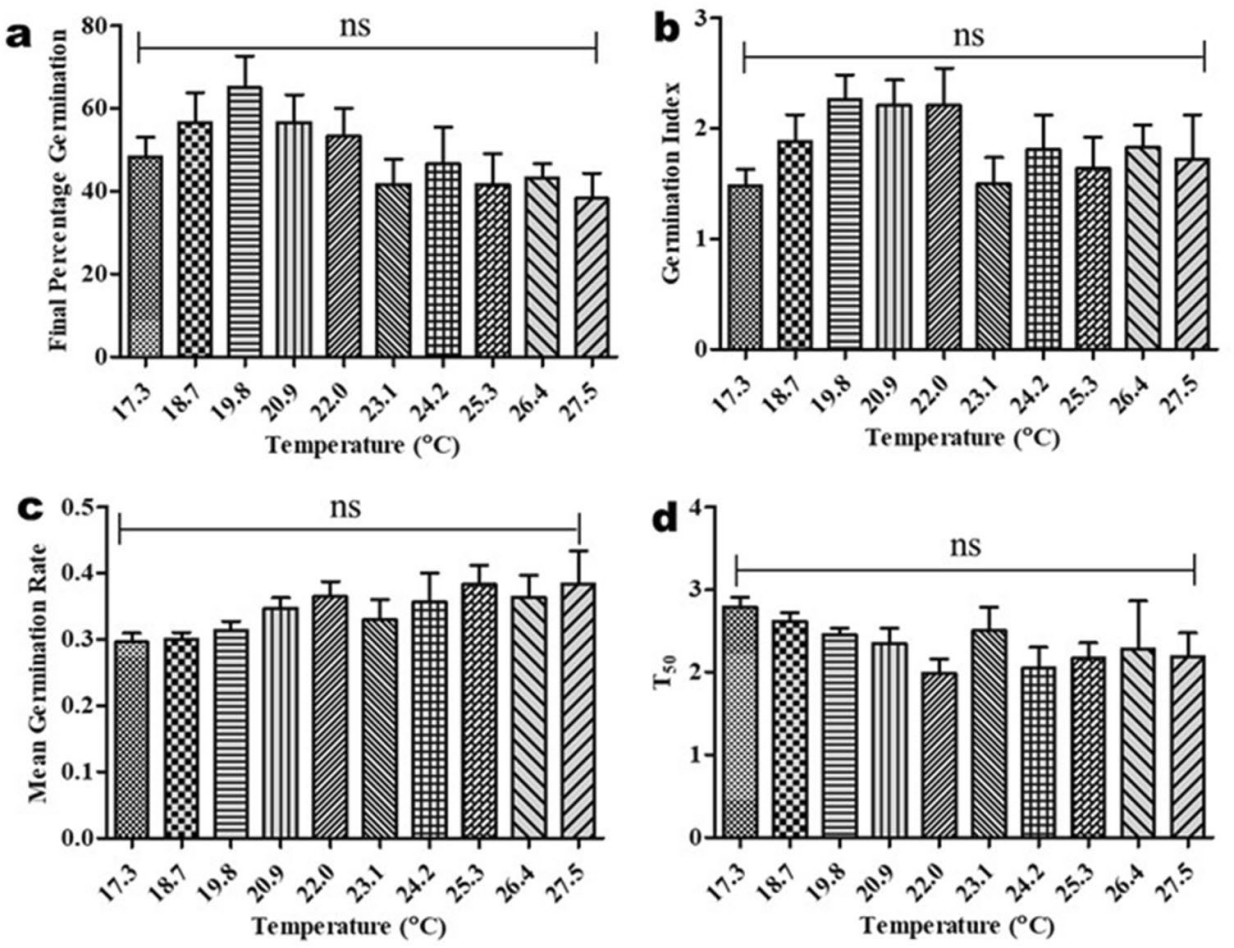
Effect of temperature on in vitro seed germination parameters of *A. africana.* (**a**) Final Percentage Germination (**b**) Germination Index (**c**) Mean Germination Rate (**d**) time required for 50% germination (T_50_). Values are presented as means ± standard error. ns-not statistically significant by Tukey’s multiple comparison test and *p* = 0.05.

With respect to FPG, the germination response of *A. africana* seeds across all temperatures was better at lower temperatures than at higher temperatures (Fig. 2a). The highest FGP and GI of 65.0 ± 7.64% and 2.26 ± 0.223, respectively, were attained at 19.8 °C, although these did not vary significantly from the values recorded at other temperatures (Fig. 2a, b). The lowest FGP (38.3 ± 6.01%) and GI (1.48 ± 0.150) values were obtained at 27.5 °C and 17.6 °C, respectively (Fig. 2a, b). *A. africana* seed germination was faster at higher temperatures than at lower temperatures with increasing MGR (highest (0.385 ± 0.050) at 27.5 °C) and decreasing T_50_ (longest (2.79 ± 0.121 days) at 17.6 °C) (Fig. 2c, d). Similar to FGP and GI, the MGR and T_50_ values across all temperatures investigated did not differ significantly (Fig. 2a–d).

### Effect of priming duration and priming treatments on in vitro germination of *A. africana* seeds

The FGP values of all primed seeds were higher than those of non-primed seeds (control) (Table 1). Among the priming treatments and across all priming durations, the highest FGPs were recorded for seeds primed with $1.44 \times 10^{ - 3 }$ M GA_3_, followed by *A. africana* seeds primed with 0.1 M KNO_3,_ and the lowest FGPs were recorded for hydro-primed seeds (Table 1). The overall highest FGP was 100 ± 0.00% for 12 h priming with $1.44 \times 10^{ - 3 } \;{\text{M}}$ GA_3_ and was significantly higher (*p* < 0.05) than other FGPs across all treatments, except for *A. africana* seeds primed in $1.44 \times 10^{ - 3 }$ M GA_3_ for 18 h (97.5 ± 2.17%) and 24 h (97.5 ± 2.50%) and 0.1 M (87.5 ± 6.29%) and 0.5 M (90.0 ± 7.07%) KNO_3_ for 6 and 12 h, respectively (Table 1). Priming duration of 12 h resulted in the highest and second highest FGPs in three (100 ± 0.00% in $1.44 \times 10^{ - 3 }$ M GA_3_, 90.0 ± 7.07% and 77.5 ± 6.29% in 0.5 and 1.0 M KNO_3_, respectively) and two (82.5 ± 2.50% in 0.1 M KNO_3_ and 65.0 ± 2.89% in H_2_O) treatments, respectively, of all seven priming treatments, and had the highest FGP among all priming durations (Table 1). Highest GI (3.80 ± 0.239) was recorded in seeds primed for 24 h in GA_3_ and this significantly differed from other GI across all treatments apart from GI for $1.44 \times 10^{ - 3 } \;{\text{M}}$ GA_3_ for 6 h (2.97 ± 0.385), 12 h (3.08 ± 0.090), and 18 h (3.25 ± 0.034); $2.89 \times 10^{ - 4 } \;{\text{M}}$ GA_3_ for 18 h (2.74 ± 0.238) and 24 h (2.99 ± 0.558); $2.89 \times 10^{ - 5 }$ M GA_3_ for 18 h (2.58 ± 0.222) and 24 h (3.31 ± 0.309); 0.1 M KNO_3_ for 6 h (2.46 ± 0.312); and H_2_O for 24 h (2.60 ± 0.417) (Table 1). In most cases, seeds primed in 1.0 M KNO_3_ had the lowest GI, with the lowest values for priming duration of 24 h (0.43 ± 0.093) (Table 1). The GI value improved with increase in priming duration for all concentrations of GA_3_ and H_2_O, but the reverse was true for KNO_3_ (Table 1). The highest MGR was recorded in seeds primed with $1.44 \times 10^{ - 3 } \;{\text{M}}$ GA_3_ for 24 h (Table 1). The T_50_ values for all concentrations of GA_3_-primed seeds decreased with an increase in priming duration (Table 1). There were no significant differences in T_50_ values across all GA_3_ priming concentrations and durations and across hydro-priming at all durations; however, these values were significantly higher (*p* < 0.05) than T_50_ for all KNO_3_ treatments, except for the treatments with 0.1 M concentrations at 6 and 18 h priming durations.

**Table 1:** Effects of different priming treatments and durations on in vitro germination of *Aspilia africana.*

Priming treatment	Duration (H)	Germination parameters			
		FGP	GI	MGR	T_50_
Non		55.0 ± 8.66 cd	1.39 ± 0.277 cdef	0.303 ± 0.027 bc	3.275 ± 0.247 bc
H_2_O	6	60.0 ± 7.07 bc	1.72 ± 0.192 cdef	0.280 ± 0.004 bc	3.044 ± 0.090 abc
	12	65.0 ± 2.89 bc	1.87 ± 0.180 cdef	0.296 ± 0.008 bc	2.990 ± 0.191 abc
	18	65.0 ± 10.41 bc	1.97 ± 0.319 bcde	0.291 ± 0.017 bc	2.948 ± 0.217 abc
	24	67.5 ± 7.50 bc	2.60 ± 0.417 abcd	0.348 ± 0.024 bc	2.167 ± 0.243 ab
0.1 M KNO_3_	6	87.5 ± 6.29 abc	2.46 ± 0.312 abcde	0.288 ± 0.009 bc	3.136 ± 0.240 bc
	12	82.5 ± 2.50 bc	2.20 ± 0.108 bcde	0.328 ± 0.036 bc	2.994 ± 0.136 abc
	18	77.5 ± 8.54 bc	2.25 ± 0.274 bcde	0.307 ± 0.018 bc	2.860 ± 0.142 abc
	24	75.0 ± 10.41 bc	2.03 ± 0.470 bcde	0.295 ± 0.019 bc	3.309 ± 0.497 bc
0.5 M KNO_3_	6	82.5 ± 8.54 bc	2.04 ± 0.234 bcde	0.302 ± 0.038 bc	3.354 ± 0.229 bc
	12	90.0 ± 7.07 abc	2.19 ± 0.344 bcde	0.274 ± 0.020 bc	3.531 ± 0.129 bc
	18	80.0 ± 7.07 bc	1.71 ± 0.204 cde	0.226 ± 0.007 c	4.142 ± 0.216 cd
	24	72.5 ± 4.79 bc	1.44 ± 0.150 de	0.265 ± 0.025 bc	4.213 ± 0.183 cd
1.0 M KNO_3_	6	62.5 ± 9.46 bc	1.43 ± 0.259 def	0.244 ± 0.011 bc	3.894 ± 0.204 cd
	12	77.5 ± 6.29 bc	1.30 ± 0.266 def	0.298 ± 0.047 bc	4.142 ± 0.618 cd
	18	67.5 ± 6.29 bc	0.96 ± 0.160 ef	0.294 ± 0.024 bc	5.063 ± 0.253 d
	24	57.5 ± 9.46 c	0.43 ± 0.093 f.	0.299 ± 0.010 bc	3.167 ± 0.687 abc
$2.89 \times 10^{ - 5 }$ M GA_3_	6	60.0 ± 0.00 bc	1.76 ± 0.106 cdef	0.315 ± 0.018 bc	2.913 ± 0.174 abc
	12	67.5 ± 2.50 bc	1.98 ± 0.083 cde	0.282 ± 0.005 bc	2.892 ± 0.116 abc
	18	72.5 ± 4.79 bc	2.58 ± 0.222 abcde	0.323 ± 0.023 bc	2.727 ± 0.338 abc
	24	75.0 ± 5.00 bc	3.31 ± 0.309 ab	0.410 ± 0.030 ab	1.700 ± 0.063 a
$2.89 \times 10^{ - 4 }$ M GA_3_	6	60.0 ± 7.07 bc	1.73 ± 0.191 cdef	0.284 ± 0.006 bc	3.044 ± 0.116 abc
	12	72.5 ± 4.79 bc	2.31 ± 0.120 cde	0.317 ± 0.006 bc	2.604 ± 0.040 abc
	18	85.0 ± 6.45 bc	2.74 ± 0.238 abcd	0.323 ± 0.024 bc	2.677 ± 0.196 abc
	24	75.0 ± 9.57 bc	2.99 ± 0.558 abc	0.407 ± 0.053 ab	2.094 ± 0.236 ab
$1.44 \times 10^{ - 3 }$ M GA_3_	6	90.0 ± 4.08 bc	2.97 ± 0.385 abc	0.318 ± 0.038 bc	2.641 ± 0.313 abc
	12	100 ± 0.00 a	3.08 ± 0.090 abc	0.318 ± 0.007 bc	2.630 ± 0.033 abc
	18	97.5 ± 2.50 ab	3.25 ± 0.034 ab	0.324 ± 0.010 bc	2.596 ± 0.086 ab
	24	97.5 ± 2.50 ab	3.80 ± 0.239 a	0.359 ± 0.018 abc	2.236 ± 0.192 ab

Means (± standard error) within a column followed by same letters are not significantly different using Tukey’s multiple comparison test and *p* = 0.05. FGP is final germination percentage, GI is germination index, MGR is mean germination rate, T_50_ is time to 50% germination.

### Effect of different priming treatments on ex vitro seed emergence

**Figure 3:**
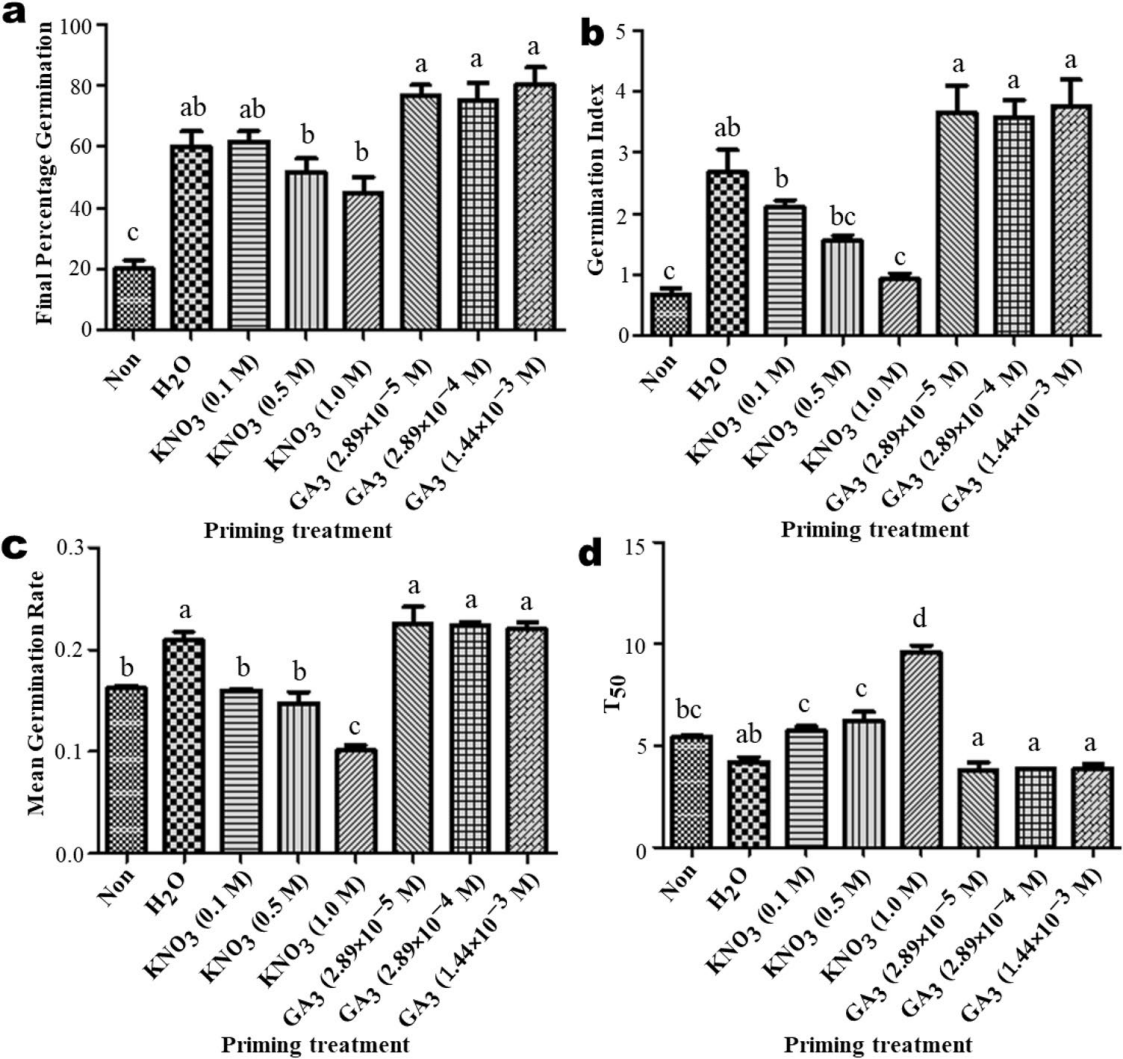
Effect of priming treatment on ex vitro seed emergence parameters of *A. africana*. (**a**) Final Percentage Germination (**b**) Germination Index (**c**) Mean Germination Rate and (**d**) time required for 50% germination (T_50_). Values are presented as means ± standard error. Same letters are not significantly different by Tukey’s multiple comparison test and *p* = 0.05.

FGPs of _GA3_-primed seeds were generally higher than those of halo- and hydro-primed seeds, with the highest overall FGP (80.0 ± 5.77%) recorded for $1.44 \times 10^{ - 3 }$ M GA_3_ primed seeds (Fig. 3a). Non-primed seeds had the lowest FGP (20.0% ± 2.89%), while the lowest FGP among primed seeds (45.0 ± 5.00%) was for the 1.0 M KNO_3_ treatment (Fig. 3a). There were no significant differences in FGP among all concentrations of GA_3_ and H_2_O and 0.1 M KNO_3_, but the FGPs of these treatments were significantly higher (*p* < 0.05) than FGPs for 0.5 M and 1.0 M KNO_3_ primed and non-primed seeds (Fig. 3a). GI in hormo-primed seeds was significantly higher (*p* < 0.05) than that in all other treatments, except for hydro-primed seeds, with the highest GI at 3.76 ± 0.434 in the $1.44 \times 10^{ - 3 }$ M GA_3_ primed seeds (Fig. 3b). GI values decreased with an increase in KNO_3_ concentration (Fig. 3b). The lowest GI value was recorded in the non-primed *A. africana* seeds (Fig. 3b). Fastest MGRs were attained in hormo-primed seeds, but did not significantly differ from MGRs of hydro-primed seeds. However, they did differ from MGRs of halo-primed and non-primed seeds (Fig. 3c). The lowest T_50_ values were recorded in GA_3_-primed seeds, followed by hydro-primed seeds (Fig. 3d). In contrast, the longest germination periods with the highest T_50_ values were for the halo-primed seeds (Fig. 3d). There were no significant differences in the T_50_ values among all hormo-primed and hydro-primed seeds (Fig. 3d). T_50_ for all hormo- and hydro-primed seeds was significantly shorter (p < 0.05) than that of all halo-primed and non-primed seeds (Fig. 3d).

### Effect of different priming treatments on the early growth of *A. africana*

**Figure 4:**
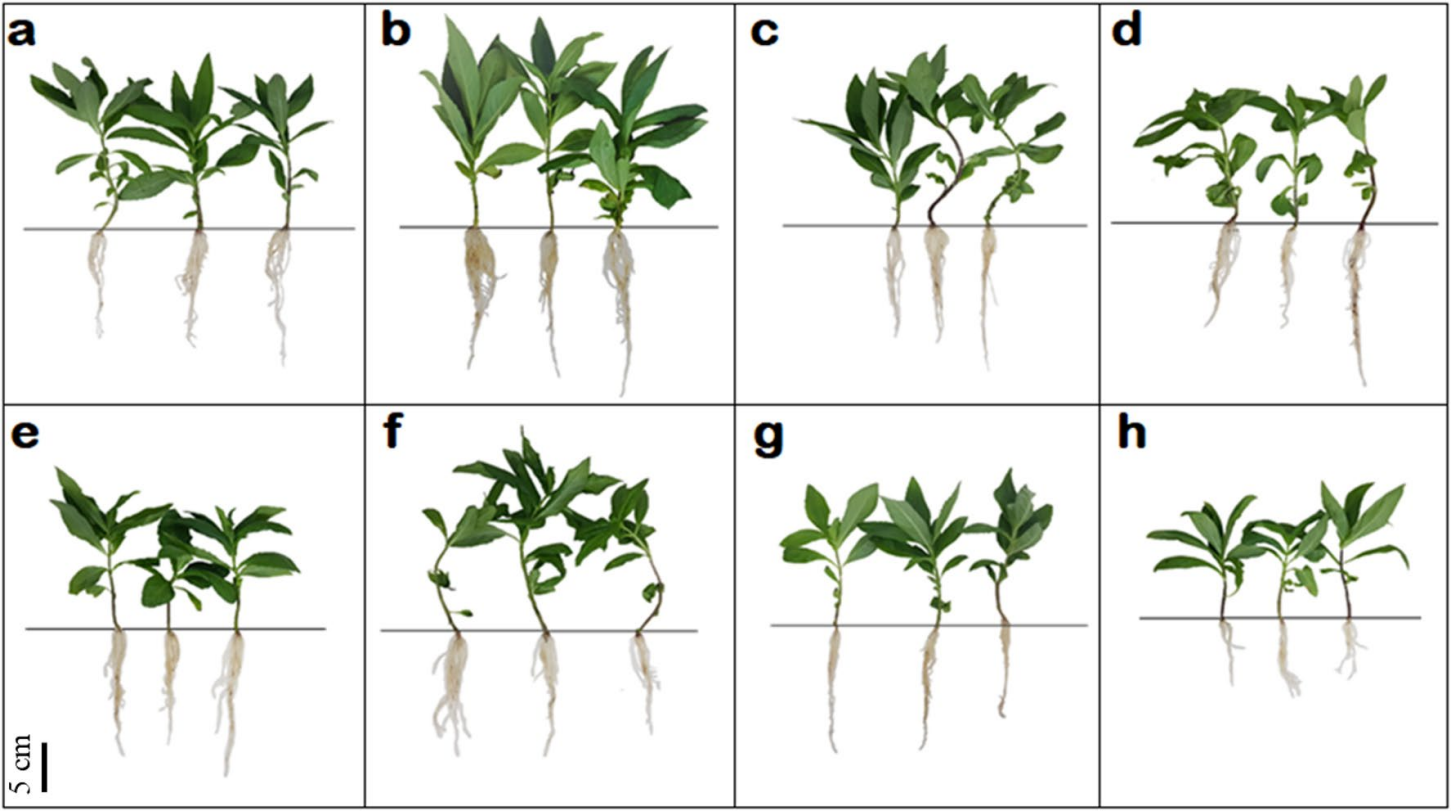
Comparison of shoot and root lengths of sampled representative *A. africana* plants derived from hormo-, halo- and hydro primed seeds after three months of growth (**a**) $1.44 \times 10^{ - 3 }$ M GA_3_ (**b**) $2.89 \times 10^{ - 4 }$ M GA_3_ (**c**) $2.89 \times 10^{ - 5 }$ M GA_3_ (**d**) 1.0 M KNO_3_ (**e**) 0.5 M KNO_3_ (**f**) 0.1 M KNO_3_ (**g**) Distilled water (**h**) Non treated.

**Figure 5:**
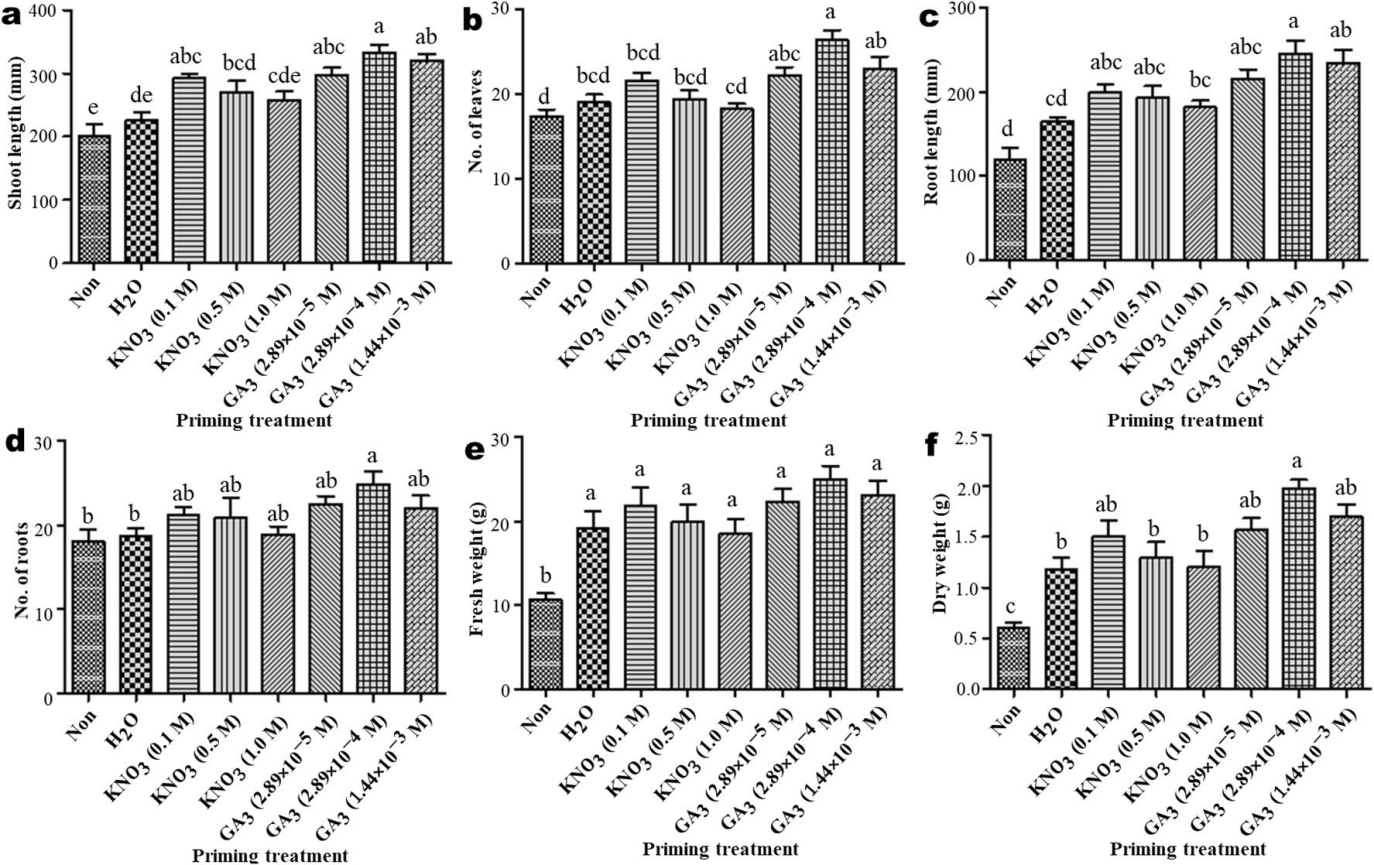
Effect of priming treatment on early growth of *A. africana.* Growth parameters measured after three months of growth. (**a**) Shoot length (**b**) Number of leaves (**c**) Root length (**d**) Number of roots (**e**) Fresh weight (**f**) Dry weight. Values are presented as means ± standard error. Same letters are not significantly different by Bonferroni’s test and *p* = 0.05.

*A. africana* plant growth was highest for seeds primed with GA_3_ followed by KNO_3_ and H_2_O, and lowest for non-primed seeds (Fig. 4). For all the growth parameters analyzed, plants from $2.89 \times 10^{ - 4 }$ M GA_3_-primed seeds exhibited the best values, whereas plants from non-primed seeds registered the lowest values for all growth parameters (Fig. 5a–f). All growth parameters of *A. africana* plants from halo-primed seeds decreased with increasing KNO_3_ concentrations (Fig. 5a–f).

The highest average shoot length (333.3 ± 11.71 mm) of *A. africana* plants from $2.89 \times 10^{ - 4 }$ M GA_3_-primed seeds did not vary significantly from the shoot lengths of plants from seeds primed with other concentrations of GA_3_ and 0.1 M KNO_3_ but significantly differed (*p* < 0.05) from the other treatments (Fig. 5a). The highest number of leaves (26.4 ± 1.15) in plants from $2.89 \times 10^{ - 4 } \;{\text{M}}$ GA_3_-primed seeds was not significantly different from that of plants from other GA_3_-primed seeds, but was significantly higher (*p* < 0.05) than those from hydro- and halo-primed seeds (Fig. 5b). Root lengths of plants from seeds primed with $2.89 \times 10^{ - 5 }$ and $1.44 \times 10^{ - 3 }$ M GA_3_, and 0.1 and 0.5 M KNO_3_ did not differ significantly from the highest average root lengths (245.0 ± 15.82) of plants from $2.89 \times 10^{ - 4 } \;{\text{M}}$ GA_3_-primed seeds, which was significantly higher (*p* < 0.05) than those from 1.0 M KNO_3_ primed, hydro-primed, and non-primed seeds (Fig. 5c). The highest number of roots (24.8 ± 1.57) from $2.89 \times 10^{ - 4 }$ M GA_3_-primed seeds did not vary significantly from those of other hormo-primed and all halo-primed seeds, but was significantly higher than those from hydro-primed and non-primed seeds (Fig. 5d). Fresh and dry weights of *A. africana* plants from all hormo-, halo-, and hydro-primed seeds were significantly higher than those from non-primed seeds (Fig. 5e, f). The fresh weights of plants from all primed seeds did not differ significantly (Fig. 5e), whereas the highest dry weight (1.98 ± 0.081 g) from $2.89 \times 10^{ - 4 }$ M GA_3_ primed seeds significantly differed from 0.5 M and 1.0 of KNO_3_ and hydro-primed seeds (Fig. 5f).

## Discussion

Temperature is a key factor that significantly affects germination^21,22^. Temperature directly influences imbibition and biochemical processes involved in germination that regulate metabolism, thus affecting germination rates and percentages^21^. Several studies have reported the effects of temperature on seed germination in different plants, including medicinal plants^23–25^. According to Baskin and Baskin^26^, the optimum temperature for many species is between 10 and 20 °C. In our study, low temperatures resulted in low FGPs and GIs for *A. africana*, and the values increased with temperature to optimal values of 65.0 ± 7.64% and 2.26 ± 0.223, respectively, at 19.8 °C, and further decreased with increase in temperature. This trend has been observed in several other medicinal plant species showing low FPG at low and high temperatures, such as *Nepeta binaludensis*, *Nepeta crassifolia*, and *Rubia tinctorum*^23^. The percentage germination linearly increases with temperature until an optimum temperature is reached, and then sharply decreases^21^. Guo, et al.^21^ further emphasizes that for most perennials, the favorable temperature for germination is 10–20 °C, and the optimum temperature for *A. africana* lies within this range. As observed, the lowest germination percentages occurred at the highest temperatures. High temperature inhibits germination of seeds in a number of species as it increases the endogenous levels of abscisic acid (ABA) by upregulating genes that biosynthesize ABA and downregulating genes associated with catabolism^27,28^. Furthermore, high temperatures decrease GA_3_ content through repression of genes that biosynthesize GA_3_, thus inhibiting seed germination^27,28^. The thermoinhibitory effect of ABA has been demonstrated in a number of plant species, including *Solanum lycopersicum*^29^, and *Pinus bungeana*^21^. The MGR and T_50_ values increased and decreased, respectively, with increasing temperature. This is presumably because the first phase of seed germination (imbibition) is greatly dependent on temperature and germination increases with increasing temperature^30^. Imbibition is a critical stage in seed germination, and the process is not only slowed down at low temperatures but also poses a great threat to cell membranes not adapted to low temperature^30^. Furthermore, the activities of some enzymes, such as dehydrogenases involved in the germination process, were found to increase with temperature^30^.

The germination parameters for primed seeds for both in vitro and ex vitro experiments were better than those for non-primed seeds. Seed priming is a simple, safe and affordable technique for improving emergence, plant growth and yield^31–33^. Seed priming reduces the effect of abiotic stress during germination leading to higher emergence of seedling and vigorous establishment of seedlings^32–34^. In line with our observations, several studies previously confirmed that priming treatments greatly improved the germination parameters in a number of plants, such as *Vicia faba* L.^35^, and lentils^36^. Seed priming improves several physiological and metabolic processes, including activation of protective enzymes, such as catalase (CAT) and superoxide dismutase (SOD), and accumulation of osmoprotectants^37^. In a study by Armin, et al.^38^, KNO_3_ treatment increased the FGP of sugar-beet seeds by up to 17.87% compared to the control. In another study, priming water melon seeds with KNO_3_ and water increased FGP and GI^39^ similar to the observations in our study. Improved germination parameters of seeds with KNO_3_ priming were also observed for *Glycine max*^40^ and *Helianthus anuus*^41^ among others. In agreement with our findings for both in vitro and ex vitro investigations, GA_3_-priming of seeds from other plants, such as *Medicago sativa*^42^, and *Hibiscus sabdariffa* L^43^. is reported to greatly improve germination.

We observed that seed germination responses to priming were in the order GA_3_ > KNO_3_ > H_2_O. Similar to our observation, in a study on the medicinal plant *Foeniculum vulgare*, it was reported that GA_3_ was also superior to other priming agents used, including KNO_3_^44^. Tahaei, et al.^44^ explained that GA_3_ improves germination by upregulating α-amylase activity, eventually improving the metabolism of starch and sugar solubility. Furthermore, GA_3_ activates embryo growth, reserve mobilization, and endosperm layer weakening, thus greatly improving germination^45,46^. Additionally, exogenous GA_3_ was observed to greatly influence radicle protrusion in germinating Arabidopsis seeds^46^. In agreement with our results, Singh et al.^47^ also observed that although both KNO_3_ and H_2_O priming of seeds improved germination parameters, FGP for KNO_3_ was better than that for H_2_O in cow pea. This could have been possible because KNO_3_ supplied nitrate to the seeds and caused exosmosis that eliminated all germination inhibiting substances^47^. A similar finding was also reported for sorghum seeds primed with KNO_3_^48^. Seed priming with KNO_3_ is known to enhance germination, improve seedling growth, seedling vigor and drought tolerance through increased water imbibition, and activation of enzymes (amylases, xylanase, and dehydrogenases) and numerous ROS-scavenging antioxidants^32^. At the imbibition stage, seeds take up increased oxygen amount, resulting in accumulation of ROS shifting the redox state^49^. KNO_3_ increases the activity of antioxidant enzymes such as SOD, CAT, ascorbate oxidase (AOX), and peroxidase (POX) in seedlings^49^.

Similar to our in vitro germination study, Damalas, et al.^35^ reported that faba bean germination parameters were affected by priming duration. In their study, hydro-priming durations of 8 and 16 h had very high FGP and GI, which declined at longer priming durations of 24 and 48 h. Contrary to their findings, in our study, seeds hydro-primed for longer durations showed slightly improved germination, but for KNO_3_ priming treatments, germination parameters declined at higher concentrations and longer treatment durations. The decline in germination in both our in vitro and ex vitro investigations with increasing concentrations of KNO_3_ was possibly due to increasing external osmotic pressure, which affected imbibition by the seeds, leading to decreased FGP, decreased GI and MGR, and a longer T_50_ duration. Oliveira, et al.^39^ also reported decreased melon seed FGP and GI with increasing salt stress. Osmotic stress affects starch hydrolysis energy production, thus affecting germination^39,50^. Furthermore, in line with our observation, Ruttanaruangboworn, et al.^51^ also reported a better germination response of *Oryza sativa L.* when primed with a lower concentration (1%) of KNO_3_ than with KNO_3_ at a higher concentration (2%). Generally, germination parameters improved with increasing GA_3_ concentration, although there were no significant differences among the GA_3_-treated seeds for both in vitro and ex vitro investigations. Increasing the concentration of GA_3_ improves the metabolic and physiological processes during germination. As in our study, priming of *Capsicum annum* L. seeds in $1.44 \times 10^{ - 3 }$ M GA_3_ resulted in the highest FGP of 85.98%^52^. Inconsistent with our findings, germination of *Leymus chinensis* seeds was best when primed with GA_3_ at a concentration of $5.05 \times 10^{ - 5 }$ M^53^. Such disparities could be attributed to differences in the species and seed conditions.

Comparing the in vitro and ex vitro germination parameters, the in vitro germination parameters were improved for both primed and non-primed *A. africana* seeds. Finch-Savage and Bassel^54^ pointed out that soil is such an intricate environment that exerts considerable stress on germinating seeds and seedlings. Seeds and seedlings are therefore vulnerable to such complexity, including mechanical impedance^54^.

*A. africana* seed priming improved plant growth for all priming solutions, with all primed seeds recording increased plant growth compared to non-primed seeds. This observation is in agreement with findings from a number of previous studies^35,39,55,56^. In fact, Zhu, et al.^57^ recorded increased root lengths, and fresh and dry stem weights of two *Brassica napus* L. varieties for all priming solutions when treated with five different priming agents that included GA_3_. Compared to non-primed seeds, priming causes increased cell division at the apical meristem of roots of seedlings, which eventually promotes growth and development^58^.

Across all measured parameters, GA_3_-primed seeds produced plants with the highest growth compared to halo- and hydro- primed seeds. These observations were similar to those of previous research findings^39,56^. The superiority of GA_3_ over halo- and hydro-priming could be because GA_3_ breaks dormancy in seeds, promoting germination, increasing intermodal lengths and cell division in the cambial zone, and also causes an increase in leaf size^56,59^.

Similar to our findings, increased growth of plants from KNO_3_ primed seeds has been previously reported^38,55,60,61^. Thejeshwini, et al.^56^ pointed out that growth of plants from KNO_3_ primed seeds was comparable to that of plants from GA_3_-primed seeds. Seed priming with KNO_3_ greatly improved soybean plant height, dry weight, seedling shoot, and root lengths^62^. In another study, KNO_3_ priming improved plant height, number of leaves, and leaf area among other growth parameters in rice^55^. Adnan, et al.^60^ explained the increased growth observed in plants from KNO_3_ primed seeds as a result of the nitrates that regulate growth and translocate photo-assimilates to specific plant parts, improving growth and yield. Hydro-priming improves the growth of a number of plants^35,60^. Hydro-priming increases shoot length, root length, and number of roots among other parameters in sorghum^60^. The shoots of hydro-primed seeds show higher amylase enzyme activity that enhances the hydrolysis of shoot transitory starch, providing more glucose and enabling more growth^58^.

## Conclusion

In this study, in vitro germination level of non-primed *A. africana* seeds was low across all investigated temperatures. Hydro-, halo-, and hormonal priming greatly improved both in vitro germination and ex vitro emergence of *A. africana* seeds. For the in vitro setup, seeds primed with $1.44 \times 10^{ - 3 }$ M GA_3_ had the highest FGP and GI, and the shortest T_50_ across all priming durations. Seeds primed in KNO_3_ had better germination parameters at shorter priming durations compared to longer priming durations. Furthermore, the highest overall FGP was observed for seeds primed for 12 h in $1.44 \times 10^{ - 3 }$ M GA_3_. Ex vitro seed emergence was significantly enhanced for seeds primed with GA_3_ compared to non-primed seeds. In addition, the ex vitro* A. africana* seed emergence was significantly enhanced with a decrease in KNO_3_ concentration. Priming *A. africana* seeds with H_2_O, KNO_3_, and GA_3_ improved their growth parameters. After three months of treatment with $2.89 \times 10^{ - 4 }$ M GA_3_, *A. africana* seeds produced plants with the longest shoot and root lengths, highest number of leaves and roots, and highest fresh and dry weights. In our study, we did not determine the base and ceiling temperatures for seed germination of *A. africana*, and we recommend further study in this regard. Seed priming of *A. africana* is a feasible approach to greatly improve germination. This is the first study investigating the effects of temperature and priming treatments on the germination and emergence of *A. africana* seeds.

## References

[1] Okello D (2021). An in vitro propagation of *Aspilia africana* (Pers.) CD Adams, and evaluation of its anatomy and physiology of acclimatized plants. Front. Plant Sci..

[2] Ajeigbe K, Onifade A, Omotoso D, Enitan S, Olaleye S (2014). Anti-ulcerogenic activity of *Aspilia africana* leaf extract: Roles of gastric acid, oxidative stress and neutrophil infiltration. Afr. J. Biomed. Res..

[3] Okoli C (2007). Anti-inflammatory activity of hexane leaf extract of *Aspilia africana* CD Adams. J. Ethnopharmacol..

[4] Okello D, Kang Y (2019). Exploring antimalarial herbal plants across communities in Uganda based on electronic data. Evid. Based Complement. Altern. Med..

[5] Okello D, Lee J, Kang Y (2020). Ethnopharmacological potential of *Aspilia africana* for the treatment of inflammatory diseases. Evid. Based Complement. Altern. Med..

[6] Niyonizigiye I (2020). Characterization and in vitro cytotoxicity of phytochemicals from *Aspilia africana* obtained using green extraction techniques. S. Afr. J. Bot..

[7] Komakech R, Matsabisa MG, Kang Y (2019). The wound healing potential of *Aspilia africana* (Pers.) CD Adams (Asteraceae). Evid. Based Complement. Altern. Med..

[8] Kumar B, Gupta E, Mali H, Singh H, Akash M (2013). Constant and alternating temperature effects on seed germination potential in *Artemisia annua* L. J. Crop Improv..

[9] Verma SK, Kumar B, Ram G, Singh H, Lal R (2010). Varietal effect on germination parameter at controlled and uncontrolled temperature in Palmarosa (*Cymbopogon martinii*). Ind. Crops Prod..

[10] Motsa MM, Slabbert M, Van Averbeke W, Morey L (2015). Effect of light and temperature on seed germination of selected African leafy vegetables. S. Afr. J. Bot..

[11] Koger CH, Reddy KN, Poston DH (2004). Factors affecting seed germination, seedling emergence, and survival of texasweed (*Caperonia palustris*). Weed Sci..

[12] Akramghaderi F, Soltani A, Sadeghipour H (2008). Cardinal temperature of germination in medical pumpkin (*Cucurbita pepo* conver. *pepo* var. *styriaca*), borago (*Borago officinalis* L.) and black cumin (*Nigella sativa* L.). Asian J. Plant Sci..

[13] Forti C (2020). Molecular dynamics of pre-germinative metabolism in primed eggplant (*Solanum melongena* L.) seeds. Hortic. Res..

[14] Du B (2019). Rice seed priming with sodium selenate: Effects on germination, seedling growth, and biochemical attributes. Sci. Rep..

[15] Rajjou L (2012). Seed germination and vigor. Annu. Rev. Plant Biol..

[16] Hussain S (2015). Benefits of rice seed priming are offset permanently by prolonged storage and the storage conditions. Sci. Rep..

[17] Oko O, Asuquo O, Agiang E, Osim E (2017). Neuroendocrine and behavioural responses of Japanese quails to dietary *Aspilia africana* leaf meal and extracts. J. Livest. Sci. (ISSN online 2277–6214).

[18] Okello D (2021). Effects of commercial soils on germination, early growth, and chlorophyll content of *Aspilia africana*, a medicinal plant. J. Plant Biotechnol..

[19] Farooq M, Basra S, Ahmad N, Hafeez K (2005). Thermal hardening: A new seed vigor enhancement tool in rice. J. Integr. Plant Biol..

[20] Reka AA (2019). Chemical, mineralogical and structural features of native and expanded perlite from Macedonia. Geol. Croat..

[21] Guo C, Shen Y, Shi F (2020). Effect of temperature, light, and storage time on the seed germination of *Pinus bungeana* Zucc. ex Endl.: The role of seed-covering layers and abscisic acid changes. Forests.

[22] Yang L-E (2020). Cold stratification, temperature, light, GA3, and KNO3 effects on seed germination of *Primula beesiana* from Yunnan, China. Plant Divers..

[23] Bannayan M, Nadjafi F, Rastgoo M, Tabrizi L (2006). Germination properties of some wild medicinal plants from Iran. Seed Technol..

[24] Kamaha C, Maguire J (1992). Effect of temperature on germination of six winter wheat cultivars. Seed Sci. Technol..

[25] Zeinati E, Soltani A, Galeshi S, Sadati S (2010). Cardinal temperatures, response to temperature and range of thermal tolerance for seed germination in wheat (*Triticum aestivum* L.) cultivars. Electron. J. Crop Prod..

[26] Baskin CC, Baskin JM (1998). Seeds: Ecology, Biogeography, and Evolution of Dormancy and Germination.

[27] Vishal B, Kumar PP (2018). Regulation of seed germination and abiotic stresses by gibberellins and abscisic acid. Front. Plant Sci..

[28] Toh S (2008). High temperature-induced abscisic acid biosynthesis and its role in the inhibition of gibberellin action in *Arabidopsis* seeds. Plant Physiol..

[29] Geshnizjani N, Ghaderi-Far F, Willems LA, Hilhorst HW, Ligterink W (2018). Characterization of and genetic variation for tomato seed thermo-inhibition and thermo-dormancy. BMC Plant Biol..

[30] Szczerba A (2021). Effect of low temperature on germination, growth, and seed yield of four soybean (*Glycine max* L.) cultivars. Agronomy.

[31] Arun MN (2022). Plant Stress Physiology-Perspectives in Agriculture.

[32] Ali LG, Nulit R, Ibrahim MH, Yien CYS (2021). Efficacy of KNO3, SiO2 and SA priming for improving emergence, seedling growth and antioxidant enzymes of rice (*Oryza sativa*), under drought. Sci. Rep..

[33] Harris D (2001). On-farm seed priming: Using participatory methods to revive and refine a key technology. Agric. Syst..

[34] Mondal, S. & Bose, B. Seed priming: An interlinking technology between seeds, Seed germination and seedling establishment. in* Plant Reproductive Ecology-Recent Advances* (ed. Rustagi, A., & Chaudhry, B.) 107–122 (IntechOpen, 2021).

[35] Damalas CA, Koutroubas SD, Fotiadis S (2019). Hydro-priming effects on seed germination and field performance of faba bean in spring sowing. Agriculture.

[36] Sağlam S, Sibel D, Gamze K, Gürbüz A (2010). Hydropriming increases germination of lentil (*Lens culinaris* Medik.) under water stress. Not. Sci. Biol..

[37] Yan M (2015). Seed priming stimulate germination and early seedling growth of Chinese cabbage under drought stress. S. Afr. J. Bot..

[38] Armin M, Asgharipour M, Razavi-Omrani M (2010). The effect of seed priming on germination and seedling growth of watermelon (*Citrullus lanatus*). Adv. Environ. Biol..

[39] da Silva Oliveira CE (2019). Seed priming improves the germination and growth rate of melon seedlings under saline stress. Ciênc. Rural.

[40] Mohammadi G (2009). The effect of seed priming on plant traits of late-spring seeded soybean (*Glycine max* L.). Am. Eurasian J. Agric. Environ. Sci..

[41] Kaya MD, Okçu G, Atak M, Cıkılı Y, Kolsarıcı Ö (2006). Seed treatments to overcome salt and drought stress during germination in sunflower (*Helianthus annuus* L.). Eur. J. Agron..

[42] Younesi O, Moradi A (2014). Effect of priming of seeds of Medicago sativa" Bami" with gibberellic acid on germination, seedlings growth and antioxidant enzymes activity under salinity stress. J. Hortic. Res..

[43] Abiri R (2016). Quantitative assessment of indica rice germination to hydropriming, hormonal priming and polyethylene glycol priming. Chil. J. Agric. Res..

[44] Tahaei A, Soleymani A, Shams M (2016). Seed germination of medicinal plant, fennel (*Foeniculum vulgare* Mill), as affected by different priming techniques. Appl. Biochem. Biotechnol..

[45] Chunthaburee S, Sanitchon J, Pattanagul W, Theerakulpisut P (2014). Alleviation of salt stress in seedlings of black glutinous rice by seed priming with spermidine and gibberellic acid. Not. Bot. Horti Agrobot. Cluj-Napoca.

[46] Gallardo K (2002). Proteomics of *Arabidopsis* seed germination. A comparative study of wild-type and gibberellin-deficient seeds. Plant Physiol..

[47] Singh A, Dahiru R, Musa M, Sani Haliru B (2014). Effect of Osmopriming duration on germination, emergence, and early growth of Cowpea (*Vigna unguiculata* (L.) Walp.) in the Sudan Savanna of Nigeria. Int. J. Agron..

[48] Singh A, Dahiru R, Musa M (2012). Osmopriming duration influence on germination, emergence and seedling growth of sorghum. Seed Technol..

[49] Hernández JA, Díaz-Vivancos P, Acosta-Motos JR, Barba-Espín GJS (2021). Potassium nitrate treatment is associated with modulation of seed water uptake, Antioxidative Metabolism and Phytohormone Levels of Pea Seedlings. Seeds.

[50] Marcos Filho, J. Fisiologia de sementes de plantas cultivadas: Fealq. *FEALQ, Piracicaba, Brazil* (2015).

[51] Ruttanaruangboworn A, Chanprasert W, Tobunluepop P, Onwimol D (2017). Effect of seed priming with different concentrations of potassium nitrate on the pattern of seed imbibition and germination of rice (*Oryza sativa* L.). J. Integr. Agric..

[52] Tombegavani SS, Zahedi B, Mousavi Fard S, Ahmadpour A (2020). Response of germination and seedling growth of pepper cultivars to seed priming by plant growth regulators. Int. J. Hortic. Sci. Technol..

[53] Ma H-Y (2018). A multi-year beneficial effect of seed priming with gibberellic acid-3 (ga 3) on plant growth and production in a perennial grass, *Leymus chinensis*. Sci. Rep..

[54] Finch-Savage WE, Bassel GW (2016). Seed vigour and crop establishment: Extending performance beyond adaptation. J. Exp. Bot..

[55] Bose B, Srivastava A, Siddique A (2016). Impact of nitrate salt hardened seeds and sowing dates on seedling stand, growth, yield attributes, nitrogen and stress metabolism of rice. Int. J. Agric. Environ. Biotechnol..

[56] Thejeshwini B, Manohar Rao A, Hanuman Nayak M, Sultana R (2019). Effect of seed priming on plant growth and bulb yield in onion (*Allium cepa* L.). Int. J. Curr. Microbiol. Appl. Sci..

[57] Zhu ZH (2021). Effects of seed priming treatments on the germination and development of two rapeseed (*Brassica napus* L.) varieties under the co-influence of low temperature and drought. Plos one.

[58] Hasanuzzaman M, Fotopoulos V (2019). Priming and Pretreatment of Seeds and Seedlings.

[59] Shukla N (2010). Effect of different concentrations of GA3 and NAA and their methods of application on growth and yield of onion (*Allium cepa* L.). Progress. Hortic..

[60] Adnan M (2020). Seed priming; An effective way to improve plant growth. EC Agric..

[61] Ghobadi M, Shafiei-Abnavi M, Jalali-Honarmand S, Ghobadi M, Mohammadi G (2012). Does KNO3 and hydropriming improve wheat (*Triticum aestivum* L.) seeds germination and seedlings growth?. Ann. Biol. Res..

[62] Ahmadvand G, Soleimani F, Saadatian B, Pouya M (2012). Effects of seed priming on germination and emergence traits of two soybean cultivars under salinity stress. J. Basic Appl. Sci. Res..

